# Role of *p73* Dinucleotide Polymorphism in Prostate Cancer and p73 Protein Isoform Balance

**DOI:** 10.1155/2014/129582

**Published:** 2014-07-06

**Authors:** L. Michael Carastro, Hui-Yi Lin, Hyun Y. Park, Donghwa Kim, Selina Radlein, Kaia K. Hampton, Ardeshir Hakam, Babu Zachariah, Julio Pow-Sang, Jong Y. Park

**Affiliations:** ^1^Department of Chemistry, Biochemistry & Physics, University of Tampa, Tampa, FL 33606, USA; ^2^Department of Biostatistics, Moffitt Cancer Center, Tampa, FL 33612, USA; ^3^Department of Cancer Epidemiology, Moffitt Cancer Center, 12902 Magnolia Drive, Tampa, FL 33612, USA; ^4^Department of Molecular Oncology, Moffitt Cancer Center, Tampa, FL 33612, USA; ^5^Department of Anatomic Pathology, Moffitt Cancer Center, Tampa, FL 33612, USA; ^6^Genitourinary Program, James Haley VA Hospital, Tampa, FL 33612, USA; ^7^Genitourinary Program, Moffitt Cancer Center, Tampa, FL 33612, USA

## Abstract

*Background*. Molecular markers for prostate cancer (PCa) risks are currently lacking. Here we address the potential association of a dinucleotide polymorphism (DNP) in exon 2 of the *p73* gene with PCa risk/progression and discern any disruption of p73 protein isoforms levels in cells harboring a *p73* DNP allele. *Methods*. We investigated the association between *p73* DNP genotype and PCa risk/aggressiveness and survival by fitting logistic regression models in 1,292 incident cases and 682 controls. *Results*. Although we detected no association between *p73* DNP and PCa risk, a significant inverse relationship between *p73* DNP and PCa aggressiveness (AT/AT + GC/AT versus GC/GC, OR = 0.55, 95%Cl = 0.31–0.99) was detected. Also, *p73* DNP is marginally associated with overall death (dominant model, HR = 0.76, 95%Cl = 0.57–1.00, *P* = 0.053) as well as PCa specific death (HR = 0.69, 95%Cl = 0.45–1.06, *P* = 0.09). Western blot analyses for p73 protein isoforms indicate that cells heterozygous for the *p73* DNP have lower levels of ∆Np73 relative to TAp73 (*P* < 0.001). *Conclusions*. Our findings are consistent with an association between *p73* DNP and low risk for PCa aggressiveness by increasing the expressed TAp73/∆Np73 protein isoform ratio.

## 1. Introduction

Prostate cancer is the most common nonskin malignancy among men worldwide. In the US, incidence rate in 2014 is 147.8 per 100,000 men per year [1]. One of the clinical challenges of prostate cancer (PCa) is distinguishing indolent from aggressive disease. This distinction is important to facilitate clinical treatment decision-making. For instance, patients with indolent disease can be classified as low risk and provided with conservative management and treatment, while patients with aggressive disease may be classified as high risk and provided with immediate therapy (surgery, radiation, and/or chemotherapy).

In the present prostate specific antigen (PSA) era, the majority of PCa cases are now diagnosed at an early stage when the tumor is confined to the prostate. Radical prostatectomy is the treatment of reference for organ-confined prostate tumors with good outcome in a large series of patients. However, about 20–30% of patients who undergo radical prostatectomy develop tumor recurrence within 10 years after surgery [2]. Currently, the level of PSA, clinical stage, and Gleason score are used to estimate prognosis and inform treatment modalities [3, 4]. Although these features are extremely useful, they do not fully account for the varied interindividual outcomes associated with treatment [5]. Therefore, there is a strong need for biomarkers that can distinguish aggressive from non-aggressive disease which is therefore of paramount importance.

Several lines of evidence support an association between a dinucleotide polymorphism (DNP) (rs1801173) in the* p73* gene and risk for several cancer types, but some studies reported conflicting results [38–41]. This particular* p73* DNP is a G4C14-to-A4T14 (*p73* DNP) (rs1801173) linked pair of transition changes located in the 5′-UTR portion of exon 2. In a meta-analysis of 8,017 various cancer patients and 10,610 controls from 27 epidemiological studies focusing on potential associations between various cancer risk and* p73* DNP, it was reported that* p73* DNP was associated with an increased risk for colorectal and head and neck cancers, but not lung, gastric, and oesophageal cancers, and PCa was not addressed [40]. In another combined analysis of 8,148 cancer patients and 8,150 controls from 26 studies, some of which overlapped with the meta-analysis, a positive association was detected between the* p73 *DNP and cervical, colorectal, head and neck, and other cancers, including breast, endometrial, non-Hodgkin's lymphoma, and ovarian cancers, but PCa was not addressed [5]. Up to this point, only one small study (*n* = 177 cases) of a population in northern India reported no association of* p73* DNP with risk of PCa [37]. Therefore, we investigated the potential for an association between risk/aggressiveness and survival of PCa and the* p73* DNP using 1,292 PCa patients, surgically treated at the Moffitt Cancer Center from 1986 to 2003, and 682 age-matched healthy male controls.

The* p73 *gene is a member of the p53 tumor suppressor family. The biology of the p73 tumor suppressor gene expression is complex and not completely understood. At least 14 isoforms of the p73 protein are translated from multiple mRNA variants transcribed from the* p73* gene [42]. These p73 protein isoforms are grouped into two major categories, TAp73 and ΔNp73, which differ in their N-termini and are transcribed from two different promoters, designated P1 and P2 (see Supplemental Figure 1 in Supplementary Material available online at http://dx.doi.org/10.1155/2014/129582). The transcriptionally active TAp73 isoforms are transcribed from the P1 promoter and include the full-length N-terminal sequence encompassing exons 1 through 3. However, the ΔNp73 isoforms are transcribed from promoter P2, which begins transcription at exon 4 (Supplemental Figure 1) and does not contain the N-terminal transactivation (TA) domain. Therefore, the ΔNp73 protein isoform acts in a dominate negative manner toward TAp73 because ΔNp73 isoforms are able to form tetramers with TAp73, as well as p53, but are not capable of activating transcription of p73- or p53-target genes [43]. This ΔNp73 dominate negative mechanism explains the observation of higher ΔNp73 levels, relative to TAp73, detected in human cancers [42, 44, 45].

The exact molecular consequences of the presence of the* p73* DNP allele are not known. Likewise, the mechanism by which the* p73* DNP influences cancer risks is also unknown. Because the* p73* DNP is located in exon 2 within the 5′-UTR of the* p73* gene and lies between the two* p73* gene promoters (Supplemental Figure 1), we speculated that this* p73* DNP might have an effect on the p73 N-terminal protein isoform balance, possibly due to the* p73* gene promoter utilization and/or stability or translational efficiency of the TAp73 mRNA or some other heretofore unknown molecular mechanism. Therefore, we investigated the relative p73 isoform protein levels in cancer cell lines and then discerned any correlation between the TAp73/ΔNp73 protein isoform ratios and the p73 DNP (rs1801173) genotype status.

## 2. Patients and Methods

### 2.1. Study Participants and Data Collection

The study population consisted of 1,292 prostatectomy cases (1,232 Caucasians and 60 African Americans) treated at the Moffitt Cancer Center from 1986 to 2003. Cases were PCa patients aged 36–84 at diagnosis with pathologically confirmed primary invasive PCa and treated with radical prostatectomy. Demographic and clinical information including age at diagnosis, Gleason score, TNM stage, and length of followup were obtained from the Moffitt Tumor Registry and medical records. Date of death, cause of death, and vital status information through September 30, 2011 were obtained from the medical records and the Moffitt Tumor Registry, which follows cases diagnosed or initially treated at the Moffitt Cancer Canter. Additional information on pretreatment serum PSA level, prostatic capsular invasion, surgical margin status, seminal vesicle invasion, and lymph node status was obtained from medical records. Recurrence was defined as elevated PSA level (>0.2 ng/mL), clinical metastasis, or PCa related death. The study was approved by the Institutional Review Board of the University of South Florida (Tampa, FL) and all participants gave a written informed consent.

Healthy controls consisted of 682 subjects (595 Caucasians and 87 African Americans) who were visiting Moffitt's Lifetime Cancer Screening Center or the James A. Haley VA Hospital (Tampa, FL). All control subjects were male and had no previous diagnosis of cancer.

### 2.2. Genomic DNA Preparation and Genotyping for* p73* DNP

Genomic DNA was prepared from either blood/buccal samples (control) or formalin-fixed paraffin embedded normal prostate tissue blocks (cases) obtained from the Tissue Core Facility at the Moffitt Cancer Center. DNA was extracted using the DNeasy tissue kit (Qiagen, Valencia, CA) according to the manufacturer's recommendations. Genomic DNA samples from cultured cell lines were extracted from cell cultures using a PureLink Genomic DNA Isolation Kit (Invitrogen-Life Technologies) according to the manufacturer's recommendations. Genomic DNA samples were quantified using a NanoDrop (Thermo Scientific). All genomic DNA samples, from patient samples and cell lines, were stored at −80°C.

The* p73* DNP was determined using a commercially available TaqMan Real-Time PCR allelic discrimination assay (Life Technologies; Assay ID number C_16180356_10) and TaqMan Gene Expression Assay Master Mix (Applied Biosystems-Life Technologies; part number 4369016) according to the manufacturer's protocol. Genotyping assays (20 *μ*L reaction volume) included 20 ng of genomic DNA and were performed using a 7900HT Fast Real-Time PCR System (Applied Biosystems-Life Technologies).

### 2.3. Cell Lines

Tissue culture cell lines HepG2, HCT116, H1299, CaCO-2, HEK293, JEKO-1, Jurkat, and BEAS-2B were acquired from ATCC. Other tissue culture cell lines used HeLa, MCF7, and H460 were kindly provided (DK). All cell lines, except H460, Jurkat, and JEKO-1, were maintained in DMEM supplemented with 10% FBS, 100 U penicillin, and 100 *μ*g/mL streptomycin. The H460, Jurkat, and JEKO-1 cell lines were maintained in RPMI supplemented with 10% heat-inactivated FBS, 100 U penicillin, and 100 *μ*g/mL streptomycin. All cell lines were cultured at 37°C and 5% CO_2_.

### 2.4. Western Analyses

Total protein samples were harvested from tissue culture cells by lysing in RIPA (Thermo Scientific) buffer supplemented with Halt Protease Inhibitor Cocktail (Thermo Scientific) according to the manufacturer's protocol. Protein samples were quantified using a BCA protein assay kit (Thermo Scientific) resolved on 10% SDS-PAGE and then electrotransferred onto Immunoblot PVDF (Bio-Rad, Hercules, CA) membranes in 1XTris-Glycine (Bio-Rad, Hercules, CA)/20% MeOH. Blotted membranes were blocked in 1XPBS/5% nonfat milk/0.1% Tween-20/0.02% NaN_3_. Blocked membranes were probed with a p73 isoform-specific primary monoclonal antibody in blocking solution at 1 : 200 (v/v) dilution or a primary polyclonal antibody against actin at 1 : 1000 (v/v) dilution for 1 hr and then were washed with three changes of blocking solution without NaN_3_ for 10 min. Primary antibodies were detected by secondary antibody-horseradish peroxidase (HRP) conjugates at 1 : 5000 dilution in blocking solution without NaN_3_ for 1 hr. After probing with secondary antibody-HRP conjugate, membranes were washed five times in 1XPBS/0.1% Tween-20 for 10 min. Immunoreactive proteins were detected by chemiluminescence using Amersham ECL Prime Western Blotting Detection reagent (GE Healthcare Life Sciences) and visualized by fluorography. The p73 isoform and actin protein bands detected by western analyses were quantified using ImageJ64 (http://rsbweb.nih.gov/ij/docs/install/osx.html), and these values were used to calculate TAp73/ΔNp73 relative expression level ratios using the formula: TAp73/ΔNp73 = ((TAp73/actin)/(ΔNp73/actin)).

### 2.5. Antibodies

Primary antibodies used to detect p73 protein isoforms were monoclonal antibody against ΔNp73 (clone 38C674; Calbiochem, Merck KGaA, Darmstadt, Germany) and monoclonal antibody against full-length TAp73 (clone 5B429; Santa Cruz Biotechnologies, Dallas, TX, USA). A goat primary polyclonal antibody was used to detect actin (I-19; Santa Cruz Biotechnologies, Dallas, TX, USA). Secondary antibody-HRP conjugates were utilized for detection of p73 isoform-specific monoclonal antibodies (Sigma-Aldrich, St Louis, MO, USA) and goat polyclonal antibody against actin (Rockland Immunochemicals, Gilbertsville, PA, USA).

### 2.6. Statistical Analysis

Descriptive statistics were used to summarize participants' demographic and clinical characteristics. The* p73* DNP genotype (rs1801173) was summarized using descriptive statistics by disease status (risk and recurrence), survival (prostate cancer specific survival and overall survival), and characteristics (Gleason score and clinical stage), the patients' outcome. Logistic regressions were applied to evaluate the* p73* DNP genotype associated with the binary outcomes, including prostate cancer risk (yes/no), recurrence (yes/no), Gleason score (8–10 versus ≤7), and TNM stage (III-IV versus I-II).

For cases, overall survival and prostate cancer specific survival were evaluated. Overall survival was defined as the time from date of surgical treatment to date of death due to any cause. For patients who do not die, time to death was censored at the time of last contact. For evaluating the DNP associated with overall survival, the overall survival curves by the genotype category were generated using the Kaplan-Meier method. The Cox proportional hazard model was used to examine the association after adjusting for age. The hazard ratios and their 95% confidence intervals will be calculated.

The competing risks approach was applied to analyze the* p73* DNP genotype associated with prostate cancer specific death. Time to prostate cancer specific death was defined as time from date of surgical treatment to date of prostate cancer specific death. Death due to other causes was treated as a competing risk. The plot of cumulative incidence of disease-specific deaths was generated. Cumulative incidence is the probability of observing a specific event at a given time-point for the individual who did not experience any event prior to this time-point. The DNP genotype associated with disease-specific death adjusting for age at diagnosis was evaluated using the competing risks regression [46]. The major allele was considered as the reference allele and all models were adjusted for age, which was age at diagnosis for cases and at enrolment for controls. Different inheritance models (log-additive and dominant) were taken into consideration. Due to a limited sample size of African Americans, we did not evaluate the recessive model.

Sample size was decided based on feasibility. The significant level of 0.05 and power of 80% were applied. With a sample size of 1,827 Caucasians (1,232 cases and 595 controls), the minimum detectable OR was 1.2. With a sample size of 147 African Americans (60 cases and 87 controls), the minimum detectable OR was 1.6.

We have assessed the potential association between p73 protein isoform ratios and the* p73* DNP genotype of cancer cell lines using the* t*-test. Deviation of genotype distribution from Hardy-Weinberg equilibrium in controls was tested using the exact test. Statistical tests were two-sided with an alpha level <0.05 considered statistically significant. All statistical analyses were performed using SAS 9.2 (SAS Institute, Cary, NC) and the cmprsk R package for the competing risk approach.

## 3. Results

Table 1 provides descriptive characteristics of the PCa patients and controls. African American patients tended to be younger than Caucasian patients (*P* < 0.001). The distribution of Gleason score, clinical stage, and proportion of recurrent cases are similar in both racial groups. As expected, means of PSA level among cases are significantly higher than controls in both races (*P* < 0.001).

### 3.1. *p73* DNP and Prostate Cancer Susceptibility and Progression

The genotype distributions of* p73* DNP among controls were consistent with Hardy-Weinberg equilibrium with a *P* value of 0.903 for Caucasians and 1.0 for African Americans using the exact test. The minor allelic frequency (MAF) of this DNP among Caucasians was similar in the public data (Table 2). A racial difference in the MAFs of* p73* DNP (rs1801173) was observed among the controls: 22% versus 11% for Caucasians versus African Americans, respectively (*P* < 0.001) (Table 2).

To determine whether this genetic variant was associated with increased risk for PCa, we compared genotype frequencies between PCa cases and controls. In both racial groups, PCa risk was not modified for subjects with* p73* DNP (additive model per AT/AT genotype, Caucasians: OR = 1.02, 95%CI = 0.86–1.21, African Americans: OR = 1.00, 95%CI = 0.49–2.07) after adjusting for age at diagnosis (Table 2).

**Figure 1:**
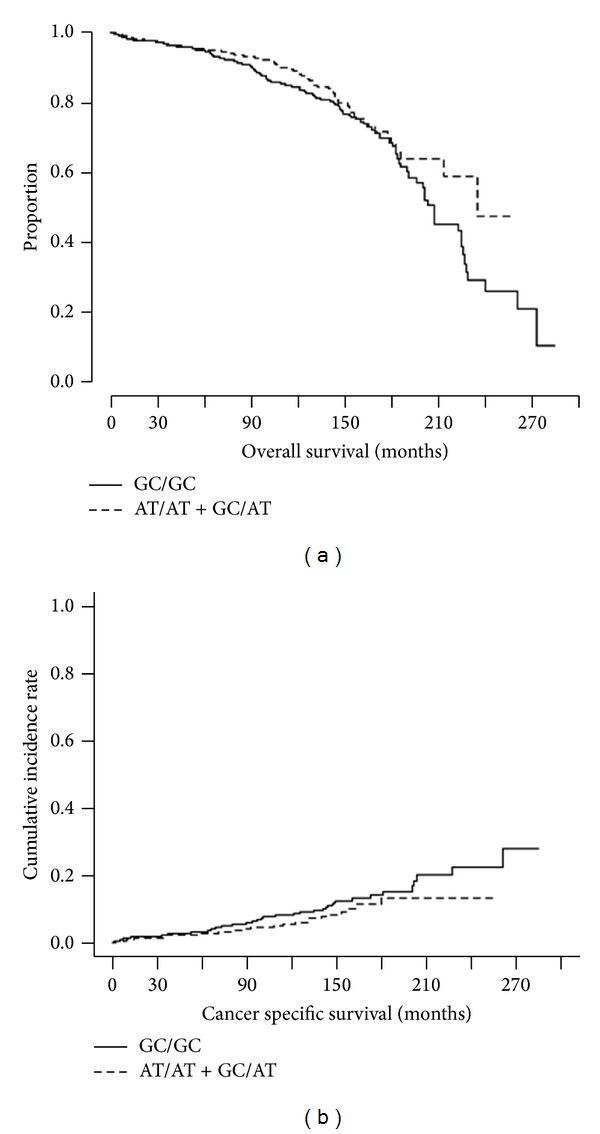
Association between rs1801173 and overall survival (a) and prostate cancer specific survival (b). A role of* p73* DNP in prostate cancer progression and aggressiveness was investigated. p73 DNP is marginally associated with overall death (dominant model, hazard ratio (HR) = 0.76, 95%CI = 0.57–1.00, *P* = 0.053 by Cox regression adjusted for age) as well as PCa specific death (HR = 0.69, 95%CI = 0.45–1.06, *P* = 0.09 by the competing risk regression adjusted for age).

We investigate a role of* p73* DNP in prostate cancer progression and aggressiveness. The* p73* DNP was significantly associated with high Gleason score [37, 40, 41] (dominant model, odds ratio (OR) = 0.55, 95% confidence interval (CI) = 0.31–0.99) among Caucasians (Table 2). The similar association was not observed in African American patients. Interestingly,* p73* DNP is marginally associated with overall death (dominant model, hazard ratio (HR) = 0.76, 95%CI = 0.57–1.00, *P* = 0.053) as well as PCa specific death (HR = 0.69, 95%CI = 0.45–1.06, *P* = 0.09, Table 2 and Figure 1). The survival analyses were not performed for African American patients due to a small sample size.

### 3.2. *p73* DNP Genotype and the p73 Protein Isoforms Ratios in Cancer Cell Lines

**Figure 2:**
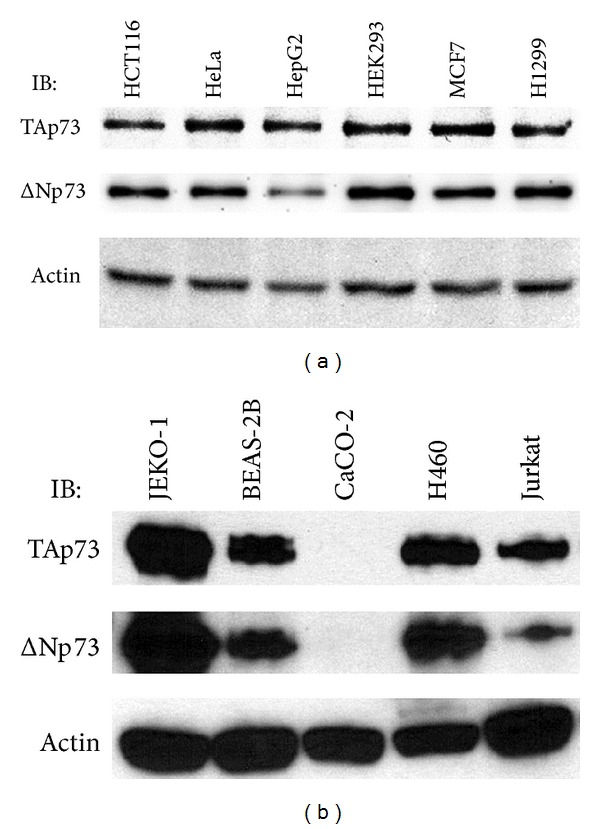
Western analyses of p73 protein N-terminal isoforms in cancer cell lines. Proteins from cancer cell line lysates in Figure 2(a) (1.25 *μ*g/well) and Figure 2(b) (7.50 *μ*g/well) were resolved on 10% SDS-PAGE gels and the resolved proteins were electrotransferred onto PVDF membranes and then immunoblotted (IB) with a p73 isoform-specific monoclonal antibody against TAp73 or ΔNp73. As a positive loading control, actin was immunoblotted with a goat polyclonal antibody. Primary antibodies were detected using appropriate secondary antibody-horseradish peroxidase conjugates and an enhanced chemoluminescence method followed by fluorography (IB = primary western antibody).

**Figure 3:**
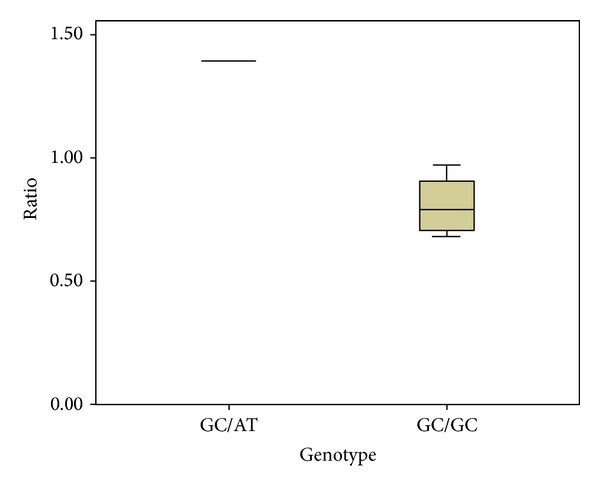
Relative TAp73/ΔNp73 protein isoform ratios in cancer cell line lysates with GC/GC and GC/AT genotype. The TAp73/ΔNp73 protein isoform levels were determined from the western blotting data in Figures 2(a) and 2(b). These p73 protein isoform levels were used to calculate the relative ratio values reported in Table 3. The mean of TAp73/ΔNp73 protein isoform ratio values is box-plotted.

We assessed the potential for a correlation between p73 protein isoform ratios and the* p73* DNP genotype of cancer cell lines observed in cultured cancer cell lines. Genomic DNA samples were isolated from eleven cultured cell lines and the* p73* DNP genotype was determined (Table 3). Total cellular protein samples were isolated from these cancer cell lines and subjected to p73 protein N-terminal isoform-specific western analyses for TAp73 and for ΔNp73, while blotting for actin in parallel as a protein loading control (Figures 2(a) and 2(b)). The TAp73 isoform, ΔNp73 isoform, and actin western blotting data were quantified using the ImageJ64 program. These values were used to calculate p73 protein N-terminal isoform ratios, in the form of TAp73/ΔNp73, relative to the actin control. Cancer cell line data were consistent with higher levels of TAp73 protein relative to ΔNp73 in the heterozygous cell lines compared to the wild type (Table 3 and Figure 3, *P* < 0.001). It was noteworthy that the only cancer cell line analyzed that was homozygous polymorphic for the* p73* DNP, CaCO-2, contained no detectable p73 protein (Figure 2(b)).

## 4. Discussion

In this study, we investigate whether the* p73* DNP is associated with modified* p73* gene expression, as well as to ascertain any association with risk/progression of PCa (Table 2). We observed that the* p73* DNP is significantly associated with high Gleason score among Caucasian men. In addition, data from our analysis of TAp73 and ΔNp73 protein isoforms in cancer cell lines is consistent with a relative increase in the ratio of TAp73/ΔNp73 protein isoforms expressed in cell lines heterozygous for the* p73* DNP compared to cell lines wild type for* p73* DNP (Table 3, Figure 3).

Multiple studies have identified the* p73* DNP as a risk factor for multiple cancer sites, including bladder [6], breast [8], cervical [12], colorectal [15], liver [26], lung [27, 28, 32], lymphoma [34], and head and neck cancers [20, 23, 24]. However, other studies have reported a decreased risk in lung [29] and head and neck cancers [19]. In addition, many studies reported no association in breast [7, 9, 10], cervical [11], colorectal [13, 14, 16], endometrial [17], head and neck [13, 18, 19, 21, 22], gastric [13, 25], ovarian [36], melanoma [35], and lung [30, 31, 33] cancers (Table 4). Recent meta-analyses have shown that the* p73* DNP is associated with various cancer risks in general [38–41]. These inconsistent results may be due to different MAFs in different ethnic populations and environmental factors. Currently, only one study (*n* = 177 cases) reported no association between PCa risk and* p73* DNP [37]. This is the first study to address the potential role for an association between aggressiveness of PCa and the* p73* DNP.

Several studies have detected a disruption in the relative abundance of the two major p73 N-terminal isoforms in tumor tissues and tumor derived cell lines when compared to normal tissues [47, 48]. The balance between TAp73 and ΔNp73 expression is altered in many cancer types due to relatively higher levels of ΔNp73 [44]. The transcriptionally inactive ΔNp73 acts in a dominate negative fashion towards TAp73 transcriptional activity and against p53 as well [44]. This dominate negative effect is thought to underlie the tumor-promoting properties associated with ΔNp73. Some reports have detected an alteration in the ratio of TAp73/ΔNp73 expression through real-time PCR analysis of mRNA and immunohistochemical staining in tumor tissues as compared to normal tissues [48, 49]. Thus, we addressed the potential for an association between* p73* DNP genotype and p73 protein isoform expression ratios through analyses of several cancer cell lines known to be p73-positve.

The* p73 *DNP, a putative functional polymorphism in exon 2, may theoretically affect a putative stem-loop structure and thereby affect p73 protein expression. Several studies have reported significant associations between the* p73* DNP (rs1801173) and various cancer types including prostate [47]. Arvanitis et al. [47] reported that the p73 protein isoform balance is disrupted in benign prostatic hyperplasia (BPH) and PCa. These results suggested that disrupted balance of p73 protein isoforms may be critical carcinogenesis in prostate.

One of the suggested biological mechanisms for the inverse relationship between the* p73* DNP allele and PCa aggressiveness comes from our p73 protein isoform molecular data. Our western analyses (Figures 2(a) and 2(b)) of multiple cancer cell lines indicate that ΔNp73 is decreased relative to TAp73 in cell lines heterozygous for* p73* DNP (Table 3 and Figure 3). Therefore, it is plausible that presence of the* p73* DNP allele causes a relative decrease in ΔNp73 protein levels in PCa tissues, which could be predicted to decrease risk for PCa aggressiveness or recurrence. Although the only cell line (CaCO-2) determined to be homozygous polymorphic for the* p73* DNP (Table 3) contained no detectable p73 protein in our western analyses (Figure 2(b)), we cannot eliminate the possibility that the CaCO-2 cell line harbors other aberrations in the* p73* alleles, such as a partial homozygous deletion as that one present in the p53-negative NCI-H1299 cell line. Further molecular studies would be needed to gain a full understanding of the molecular mechanism(s) by which the* p73* DNP genetic variant might result in lower expression levels of ΔNp73 relative to TAp73 in PCa.

The limitations of the current study are a small sample size of African American population and cancer cell lines. Therefore, a larger study is necessary to confirm this observed association between protein expression and risk/progression of PCa and* p73* DNP is real and not a chance finding. Additionally, future studies of p73 protein isoform ratios in cancer cell lines derived from prostate cancer tissues may provide further insight into the relationship between* p73* DNP genotype and p73 protein isoform expression levels.

In summary,* p73* DNP may influence aggressiveness of prostate cancer and p73 protein expression. These novel findings warrant a larger study to investigate their significance. The identification of the* p73 *DNP allele as a protective factor for aggressiveness of PCa could lead to personalized intervention strategies, which can be applied that are appropriate for the level of risk.

## Supplementary Material

Supplement Figure 1. *p73* Gene and N-terminal isoforms of *p73* protein. The structure of the *p73* gene is depicted, including the *p73* gene exons (black filled boxes for all exons, except exon 1 and exon 4 are labeled unfilled boxes), introns (lines connecting boxes), and *p73* gene promotes (P1 & P2). The approximate position of the p73 dinucleotide polymorphism (*p73* DNP) in exon 2 is indicated. The two major N-terminal protein isoforms of *p73*, TAp73 and ΔNp73, are depicted (below the *p73* gene), including the major functional domains (TA = transactivation domain; DBD = DNA-binding domain; OD = oligomerization domain). The TAp73 form includes exons 1 through 3, which are absent in the ΔNp73 isoforms.

## Figures and Tables

**Table 1:** Characteristics of study subjects by disease status and data source.

		Caucasians, *N* (%)		African Americans, *N* (%)	
		Cases, (*n* = 1232)	Controls, (*n* = 595)	Cases, (*n* = 60)	Controls, (*n* = 87)
Age^1^	Mean ± SD	60.0 ± 7.4	61.3 ± 9.6	56.4 ± 7.4	57.3 ± 9.2

Gleason score	≤6	628 (60.6)	N/A	34 (60.6)	N/A
	7	348 (33.6)		19 (33.9)	
	8–10	60 (5.8)		3 (5.3)	

Stage	1	55 (4.5)	N/A	2 (3.4)	N/A
	2	955 (78.3)		48 (81.4)	
	3	201 (16.5)		9 (15.2)	
	4	9 (0.7)		0 (0.0)	

PSA level		7.5 ± 9.1	1.1 ± 1.1	5.1 ± 3.1	1.3 ± 1.4

Recurrence	No	905 (73.6)	N/A	43 (71.7)	N/A
	Yes	325 (26.4)		17 (28.3)	

Death	Alive	975 (79.9)		54 (90.0)	
	PCa death	106 (8.7)		2 (3.3)	
	Other death cases	139 (11.4)		4 (6.7)	

Marital status	Divorced	80 (6.6)		5 (8.3)	
	Married	1057 (87.0)		49 (81.7)	
	Separated	4 (0.3)		1 (1.7)	
	Widowed	21 (1.7)		0 (0)	
	Single	53 (4.4)		5 (8.3)	

Weight (kg)		88.4 ± 14.6	93.6 ± 21.6	90.5 ± 14.2	92.5 ± 18.7

Height (m)		1.76 ± 0.07	1.76 ± 0.18	1.77 ± 0.06	1.75 ± 0.21

^1^Age at diagnosis for cases and age at enrolment for controls.

**Table 2:** Associations of genotypes with prostate cancer risk, aggressiveness, and survival.

Factors	Race	GC/GC	GC/AT	AT/AT	Dom^1^ OR (95% CI)^2^	*P* value	Additive^1^ OR (95% CI)^2^	*P* value
Case/control	White	750/357	417/202	65/27	1.00 (0.82–1.23)	0.995	1.02 (0.86–1.21)	0.82
	Black	49/68	9/16	2/1	0.87 (0.37–2.04)	0.75	1.00 (0.49–2.07)	0.99
Recurrence Y/N	White	205/544	100/316	20/45	0.86 (0.66–1.12)	0.25	0.92 (0.74–1.15)	0.48
	Black	14/35	3/6	0/2	0.97 (0.21–4.36)	0.97	0.76 (0.22–2.69)	0.68
Gleason 8–10/≤7	White	44/589	14/336	2/51	**0.55 (0.31**–**0.99)**	**0.04**	0.61 (0.37–1.02)	0.06
	Black	2/43	1/8	0/2	2.16 (0.17–26.99)	0.55	1.36 (0.19–9.92)	0.76
Stage 3 & 4/1 & 2	White	129/615	70/342	11/53	0.97 (0.71–1.31)	0.83	0.98 (0.76–1.26)	0.85
	Black	8/40	1/8	0/2	0.46 (0.05–4.63)	0.51	0.43 (0.06–3.07)	0.40

					HR (95% CI)^3^	*P* value	HR (95% CI)^3^	*P* value

Overall death Death/alive	White	160/590	68/348	17/48	**0.76 (0.57**–**1.00)**	**0.05**	0.87 (0.68–1.10)	0.24
	Black	5/44	1/8	0/2	—	—	—	—
Specific death Other/PCa/alive	White	88/72/590	40/28/338	11/6/47	0.84 (0.58–1.21)^4^	0.35	0.93 (0.67–1.28)^4^	0.64
					0.69 (0.45–1.06)^5^	0.09	0.81 (0.55–1.20)^5^	0.29
	Black	3/2/44	1/0/8	0/0/2	—	—	—	—

^1^Dom: dominant (AT/AT + GC/AT versus GC/GC); add: log-additive (per AT/AT).

^2^OR: odds ratio; CI: confidence interval; logistic model adjusted for age.

^3^HR: hazard ratio; for overall death, Cox model; for specific death, competing risk regression; all models adjusted for age.

^4^Other death cases versus alive.

^5^PCa death versus alive.

**Table 3:** *p73* DNP genotypes and *p73* isoform expression ratios for cancer cell lines.

Cell line	*p73* DNP genotype	TAp73/ΔNp73*
HCT116	GC/GC	0.68
HeLa	GC/GC	0.95
H460	GC/GC	0.74
HEK293	GC/GC	0.72
MCF7	GC/GC	0.97
H1299	GC/GC	0.69
JEKO-1	GC/GC	0.84
BEAS-2B	GC/GC	0.86
Jurkat	GC/AT	1.40
HepG2	GC/AT	1.39
CaCO-2	AT/AT	N/A

Cell lines were used for genotyping for *p73* DNP status and western analyses for *p73* protein N-terminal isoforms and beta-actin. ∗TAp73/ΔNp73 protein isoform levels were determined from the western data in Figures 2(a) and 2(b) by quantifying protein bands using ImageJ64. Relative TAp73/ΔNp73 protein isoform ratios were corrected for using actin levels as loading controls and were calculated using the formula: TAp73/ΔNp73 = ((TAp73/actin)/(ΔNp73/actin)).

**Table 4:** Summary of association studies of *p73* DNP in various cancer sites^1^.

Site	Population	Case/control	OR (95% CI)	Reference
Bladder	Indian	200/200	**1.54 (1.02**–**2.33)**	[6]

Breast	Chinese	170/178	0.77 (0.50–1.19)	[7]
Breast	Chinese	170/0	**2.76** (1.17–6.49)^2^	[8]
Breast	Japanese	200/282	0.82 (0.57–1.19)	[9]
Breast	France white	59/34	2.46 (0.92–6.58)	[10]

Cervical	Portuguese	176/141	1.01 (0.63–1.62)	[11]
Cervical	Japanese	112/442	**1.51 (1.00**–**2.30)**	[12]

Colorectal	Japanese	147/235	0.85 (0.56–1.29)	[13]
Colorectal	Tunisian	150/204	1.09 (0.71–1.66)	[14]
Colorectal	Korean	383/469	**1.50 (1.14**–**1.96)**	[15]
Colorectal	Swedish	179/260	0.92 (0.53–1.53)	[16]

Endometrial	Japanese	114/442	1.36 (0.90–2.06)	[17]

Esophageal	Chinese	348/630	1.02 (0.78–1.34)	[18]
Esophageal	Japanese	102/235	0.64 (0.40–1.04)	[13]
Esophageal	UK whites	84/152	0.90 (0.53–1.53)	[19]
H&N	US white	708/1229	**1.31 (1.09**–**1.58)**	[20]
H&N	Italian	283/295	1.36 (0.95–1.93)	[21]
H&N	US whites	326/349	1.06 (0.78–1.45)	[22]
Oral	Indian	303/319	**2.38 (1.73**–**3.29)**	[23]
Oropharyngeal	US mixed	309/0	**2.10** (1.20–3.80)^2,3^	[24]

Gastric	Italian	114/295	0.94 (0.58–1.54)	[25]
Gastric	Japanese	144/235	0.88 (0.58–1.34)	[13]

Liver	Chinese	476/526	**2.19** (1.25–3.83)^4^	[26]

Lung	Chinese	293/380	**1.48 (1.08**–**2.02)**	[27]
Lung	US white	1054/1139	**1.34 (1.13**–**1.59)**	[28]
Lung	Chinese	425/588	**0.67 (0.52**–**0.86)**	[29]
Lung	Korean	582/582	1.13 (0.90–1.43)	[30, 31]
Lung	US white	863/852	**1.34 (1.10**–**1.64)**	[32]
Lung	Japanese	189/235	0.91 (0.62–1.34)	[33]

Lymphoma	Japanese	103/440	**1.61 (1.04**–**2.47)**	[34]

Melanoma	Whites	805/838	1.05 (0.86–1.28)	[35]

Ovary	Chinese	257/257	0.81 (0.95–1.18)	[36]

Prostate	Indian	177/265	1.21 (0.80–1.86)	[37]

^1^Analysis is based on dominant model (AT/AT + GC/AT versus GC/GC).

^2^Cases only.

^3^HPV16+ versus HPV16−.

^4^HbsAg-positive individuals.
