# Supplementary material for: Role of p73 Dinucleotide Polymorphism in Prostate Cancer and p73 Protein Isoform Balance
Source: Prostate Cancer. 2014 Jul 6;2014:129582. doi: 10.1155/2014/129582 (PMC4109114; doi:10.1155/2014/129582)

Supplement Figure 1. *p73* Gene and N-terminal isoforms of p73 protein. The structure of the *p73* gene is depicted, including the *p73* gene exons (black filled boxes for all exons, except exon 1 and exon 4 are labeled unfilled boxes), introns (lines connecting boxes), and *p73* gene promoters (P1 & P2). The approximate position of the *p73* dinucleotide polymorphism (p73 DNP) in exon 2 is indicated. The two major N-terminal protein isoforms of p73, TAp73 and ΔNp73, are depicted (below the *p73* gene), including the major functional domains (TA = transactivation domain; DBD = DNA-binding domain; OD = oligomerization domain). The TAp73 form includes exons 1 through 3, which are absent in the ΔNp73 isoforms.

Supplemental Figure 1. *p73* Gene and N-terminal isoforms of p73 protein.

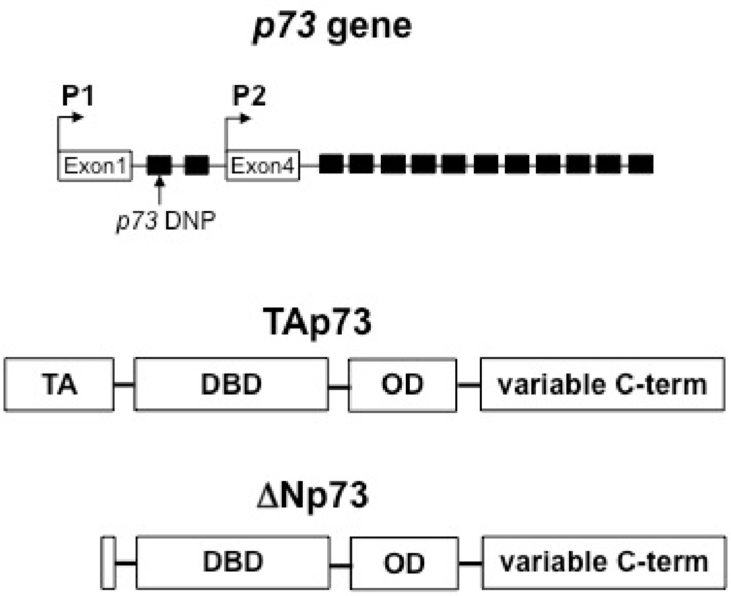

Supplement: Supplementary file 1 — Supplement Figure 1. p73 Gene and N-terminal isoforms of p73 protein. The structure of the p73 gene is depicted, including the p73 gene exons (black filled boxes for all exons, except exon 1 and exon 4 are labeled unfilled boxes), introns (lines connecting boxes), and p73 gene promotes (P1 & P2). The approximate position of the p73 dinucleotide polymorphism (p73 DNP) in exon 2 is indicated. The two major N-terminal protein isoforms of p73, TAp73 and ΔNp73, are depicted (below the p73 gene), including the major functional domains (TA = transactivation domain; DBD = DNA-binding domain; OD = oligomerization domain). The TAp73 form includes exons 1 through 3, which are absent in the ΔNp73 isoforms. [file 129582.f1.pdf]
